# Prevalence and co-occurrence of lifestyle risk factors for non-communicable diseases according to sociodemographic characteristics among adults Chilean residents

**DOI:** 10.1038/s41598-021-01167-9

**Published:** 2021-11-04

**Authors:** María José Aburto, Dayna Romero, Leandro F. M. Rezende, Zila M. Sanchez, Cristian Cofre Bolados, Juan Guzmán-Habinger, Mario Rios, Mónica Suárez-Reyes, Adilson Marques, Clemens Drenowatz, Carlos Cristi-Montero, Gerson Ferrari

**Affiliations:** 1 Universidad de Santiago de Chile (USACH), Escuela de Ciencias de la Actividad Física, el Deporte y la Salud, Las Sophoras 175, Estación Central, Santiago, Chile; 2 Departamento de Medicina Preventiva, Escola Paulista de Medicina, Universidade Federal de São Paulo, São Paulo, Brazil; 3 Universidad Mayor, Facultad de Ciências, Santiago de Chile, Chile; 4 Especialidad medicina del deporte y la actividad física, Universidad Mayor, Facultad de Ciencias, Santiago de Chile, Chile; 5 CIPER, Faculdade de Motricidade Humana, Universidade de Lisboa, 1499-002 Lisbon, Portugal; 6 ISAMB, Faculdade de Medicina, Universidade de Lisboa, 1649-028 Lisbon, Portugal; 7 Division of Sport, Physical Activity and Health, University of Education Upper Austria, 4020 Linz, Austria; 8 IRyS Group, Physical Education School, Pontificia Universidad Católica de Valparaíso, Valparaiso, Chile

**Keywords:** Cardiovascular diseases, Nutrition disorders

## Abstract

To examine the prevalence and co-occurrence of lifestyle risk factors for non-communicable diseases (NCDs) according to sociodemographic characteristics in Chilean residents. A cross-sectional study based on data from 5995 adults from the Chilean National Health Survey. The lifestyle risk factors included were physical inactivity, tobacco consumption, alcohol consumption, low fruits and vegetable consumption, and overweight/obesity. The most frequent risk factor was overweight/obesity (75.6%), followed by alcohol consumption (74.8%), low fruits and vegetable consumption (51.7%), physical inactivity (36.3%), and tobacco consumption (27.9%). Only 1.0% of the participants did not present any risk factor, while 9.6%, 30.4%, 34.0%, 20.3%, and 4.7% accumulated one, two, three, four, and five risk factors. Men (OR 1.56; 95% CI 1.18; 2.04), people who have secondary education (OR 1.59; 95% CI 1.20; 2.10), and those with lower household income (OR 1.39; 95% CI 1.09; 1.59) had higher odds of three or more risk factors. Associations were inverse for older adults (OR 0.57; 95% CI 0.41; 0.79) and rural geographic areas (OR 0.77; 95% CI 0.67; 0.89). The prevalence of risk factors for NCDs is fairly high in Chilean residents. Interventions may need to target these co-occurrences rather than emphasizing individual risk factors for NCDs. Interventions could further consider these co-occurrences as a potential target for population stratification.

## Introduction

Non-communicable diseases (NCDs) are the main cause of death worldwide^1–3^. The prevalence of NCDs, such as cardiovascular disease, cancer, diabetes, and respiratory diseases continues to increase in all age groups from 2005 to 2015 in the Americas region^4,5^. In Chile, NCDs are considered a significant challenge for development in the twenty-first century, since they contribute 58% of the premature deaths in the country^6–8^.

It is well known that physical inactivity, tobacco use, alcohol consumption, unhealthy diet, and overweight/obesity are modifiable risk factors for NCDs^9–11^. Around three in ten of all cancer cases and 36% of all cancer deaths in Chilean adults in 2018 were attributable to lifestyle risk factors. Smoking and high body mass index (BMI) were the leading causes of preventable cancers, followed by alcohol consumption, physical inactivity, low consumption of fruits and vegetables and passive smoking^12^.

Over the past two decades, Chile has undergone important health and epidemiological transitions. The prevalence of overweight/obesity reached 78% in 2017, among which, approximately, 4 million inhabitants (≥ 15 years) were living with obesity^13^. Additionally, 24% of the inhabitants are considered to be physically inactive^13^. Recently, Chile has experienced fast socioeconomic growth and lifestyle transition^14,15^, leading to the Chilean population having the highest prevalence of lifestyle risk factors for NCDs, particularly overweight and obesity, across Latin American countries^16–18^.

A recent study has indicated that the prevalence of NCDs has grown significantly in Chile between 1990 and 2019^10^. Nonetheless, this study did not provide details on the prevalence and co-occurrence of lifestyle risk factors for NCDs in Chilean residents. Monitoring lifestyle risk factors for NCDs in adults, including their co-occurrence in the population, is important as risk factors can interact with each other, thereby producing greater risk of NCDs than the sum of individual risks^19,20^. In addition, co-occurrence of lifestyle risk factors has been associated with an increase in risk of all-cause and cardiovascular disease mortality^21^. This kind of evidence is crucial at the public health level to address comprehensive interventions and reduce the burden of diseases due to the synergistic association among risk factors. Such information could inform future public health policies and interventions aiming to reduce the burden of NCDs in Chile.

In this study, we aimed to describe the prevalence and co-occurrence of lifestyle risk factors (physical inactivity, tobacco, alcohol, fruit and vegetable consumptions, and overweight/obesity) for NCDs in Chilean residents. We also examined the association between co-occurrence of three of more lifestyle risk factors and sociodemographic characteristics.

## Methods

### Chilean National Health Survey

This cross-sectional study was based on data from participants from the 2016–2017 Chilean National Health Survey (CNHS, *Encuesta Nacional de Salud*). The CNHS is a nationally representative population-based household survey on health behaviors^13^. A stratified multistage probability sample of 6233 participants aged ≥ 15 years was recruited. The sample included non-institucionalized participants (Chilean or foreign), located in urban and rural areas of the fifteen regions of Chile. Through the Kish algorithm, one participant per household was randomly selected. Response rate was 67%, with no replacements. In our study, we excluded adolescents between 15 to 17 years of age due to small sample size to provide specific analysis for this group. Of note, adolescents have different lifestyle behaviors than those observed in adults^22,23^. Additionally, participants with missing data on any lifestyle risk factors (n = 238) were excluded.Thus, the final analytical sample included 5995 participants between 18 and 98 years of age with complete data.

Data was collected through structured face-to-face interviews carried out in the households between August 2016 and March 2017. The main survey included questions covering health conditions, health risk and protective factors (e.g. physical activity, tobacco use, alcohol use, overweight). The interviewers collected anthropometric measurements and self-reported information on health, household characteristics, and living conditions.

The study protocol was approved of the National Health Survey of Chile 2016–2017 and by the Scientific Ethics Committee of the Faculty of Medicine of Pontificia Universidad Católica de Chile (project number 16-019), and written informed consent was obtained before data collection. All aspects of the study were in accordance with the Declaration of Helsinki.

### Lifestyle risk factors for non-communicable disease

The lifestyle risk factors for NCDs included in this study were physical inactivity, tobacco use, alcohol use, low intake of fruits and vegetables and overweight/obesity.

Physical inactivity was assessed using the Global Physical Activity Questionnaire (GPAQ)^24,25^. Participants informed the duration, frequency, and intensity of physical activities performed in three different domains (occupational, active commuting, and recreational). For each domain, we assigned metabolic-equivalent of tasks (MET; where 1 MET =  ~ 3.5 ml O_2_ kg^−1^ min^−1^) according to the GPAQ protocol (4-METs was used for moderate and transport-related activities and 8-METs for vigorous activities). Total self-reported physical activity was calculated as the sum of MET-min/week^−1^ across all three domains, and participants were subsequently categorized as physically inactive (< 600 MET-min/week^−1^) or active (≥ 600 MET-min/week^−1^)^26^.

Tobacco smoking was derived from the following question: “Currently, do you smoke cigarettes?” (occasionally, < 1 cigarette per day, > 1 cigarette per day, quitted smoking, never smoked). We grouped the first three categories in smokers and the last two in never/former smoker)^13,27,28^.

Alcohol consumption was estimated by the short version of the Alcohol Use Disorder Identification Test (AUDIT-C), which was developed by the World Health Organization^29^, adapted and validated for use in Chile^30^. The question “Did you drink alcohol at least once last month?” was used to examine current alcohol consumption^31^. A positive answer to this question was considered alcohol use and, therefore, a risk factor for NCDs^31^.

The consumption of fruits and vegetables was estimated through the questions: “Typically, how many days a week do you eat fruits?” and “Typically, how many days a week do you eat vegetables or vegetables salad?” (do not include legumes or potatoes), respectively^13,32^. We considered low consumption of fruits and vegetables as four or fewer sum days per week^33–35^.

The BMI was obtained trough using objectively measures of height and weight. A portable square was used to mark the height on a wall, that was later measured with a metal tape (nearest 0.1 cm). The body weight was measured using a digital scale (OMRON HN 289) to the nearest 0.1 kg. Weight measurements were taken with participants in bare feet and light clothing^13^. BMI was calculated as weight/height^2^ and participants were classified as normal weight [≤ 24.9 kg/m^2^] or overweight/obese [≥ 25.0 kg/m^2^])^13^.

### Sociodemographic characteristics

Sociodemographic information included sex (women/men), age (younger [18–36 years], middle-age [37–56 years], and older adults [> 56 years]), education level (up to primary [< 8 years of studies], secondary [between 8 and 12 years of studies] and beyond secondary [> 12 years of study]), monthly household income (stratified into tertiles: lowest [< US $310.00], medium [US $310.00–705.00], and highest [> US 705.00]), health insurance (private [Isapres] and public [Fonasa]), and indigenous ethnicity (yes/no). In Chile, there are two main ethnicities; the first relates to Indigenous, and the second relates to those with other roots^36^. We also considered urban–rural geographic areas based on the Chilean population census^13^.

### Statistical analysis

Prevalences and 95% confidence intervals (95% CI) were computed to describe the ocurrence and co-occurrence of risk factors for NCDs according to sociodemographic characteristics. In the descriptive analysis, the comparisons were made by analyzing the overlaps of 95% CI, with a significant difference being considered when there was no overlap of the 95% CI; and no difference was considered when one of the 95% CI was partially included by the other^37^.

Thirty-two possible combinations of the five risk factors were considered (all potential combinations of five yes–no variables = 2^5^). Co-occurrence of risk factors was considered as the observed prevalence of combined risk factors higher than the expected prevalence if risk factors were independent, i.e. the observed/expected ratio prevalences was > 1^38^. The presence of three or more risk factors was considered as the threshold for identifying a co-occurrence phenomenon because, after this threshold, the rates dropped down suddenly. The expected prevalence was calculated by multiplying the marginal (raw) prevalence of individual risk factors. A multivariable logistic regression model was performed to examine the odds ratio (OR) and its 95% CI for the association between sociodemograpic characteristics (sex, age, education level, household income, health insurance, ethnicity, and the geographic area—mutually adjusted for each other) and three or more risk factors.

Analyses were weighted by the survey design^13^. All statistical analyses were conducted using SPSS V22 software (SPSS Inc., IBM Corp., Armonk, New York, NY, USA). Weights took into account the complex survey design, and the four levels of the multistage sampling.

## Results

The total number of participants included in the study was 5995 (3796; 61.5% women) with a mean age of 50.2 years (95% CI 48.4; 52.0). Overall, 29.6% were middle-aged (37–56 years), 53.3% had between 8 to 12 years of education, 40.8% were in the middle tertile of household income, 73.2% had public health insurance, 88.1% were not of indigenous heritage, and 84.1% lived in urban areas.

Table 1 presents the characteristics of the participants. Overall, the most frequent risk factor was overweight/obesity (75.6%), followed by alcohol consumption (74.8%), low fruits and vegetable consumption (51.7%), physical inactivity (36.3%), and tobacco consumption (27.9%). Men had higher prevalence of tobacco smoking, alcohol consumption, low fruits and vegetable consumption, and overweight/obesity than women. The younger age group (< 37 years) presented a higher prevalence of physical inactivity and low consumption of fruits and vegetables. For tobacco smoking and alcohol consumption, the prevalence did not differ between the first two age groups. Overweight/obsesity was more prevalent in the middle age. Participants with lower education level (up to primary) and household income (first tercile) showed lower prevalence of tobacco smoking and alcohol consumption, while there was no difference in physical inactivity. Overweight/obesity was more prevalent among the lower education level participants, but no differences were found in the different categories of household income. Compared to participants with public health insurance, participants with private health insurance had higher prevalence of alcohol consumption, but lower prevalence of physical inactivity and overweight/obesity. Participants who lived in urban areas had higher prevalence of tobacco smoking and a lower prevalence of overweight/obesity compared to those living in the rural areas (Table 1).

**Table 1:** Prevalence and 95% confidence intervals of risk factors for non-communicable disease in Chilean residents.

Sociodemographic characteristics	Physical inactivity	Tobacco consumption	Alcohol consumption	Low fruits and vegetables consumption	Overweight/obesity
	% (95% CI)	% (95% CI)	% (95% CI)	% (95% CI)	% (95% CI)
Overall	36.3 (35.2; 37.5)	27.9 (26.6; 29.0)	74.8 (73.3; 76.2)	51.7 (50.4; 53.0)	75.6 (74.5; 76.8)
**Sex**					
Women	36.1 (34.7; 37.7)	25.4 (24.0; 26.9)	69.5 (67.6; 71.5)	48.4 (47.0; 50.1)	76.7 (75.3; 78.1)
Men	36.5 (34.5; 38.7)	32.1 (30.2; 34.0)	81.4 (79.6; 83.4)	57.1 (55.2; 59.0)	73.8 (71.8; 75.7)
**Age group**					
Younger (18–36 y)	40.6 (38.4; 42.9)	35.9 (33.8; 38.0)	77.5 (75.0; 79.6)	62.0 (59.9; 64.2)	64.1 (61.7; 66.3)
Middle age (37–65 y)	33.1 (31.0; 35.1)	32.9 (30.7; 35.1)	76.7 (74.3; 79.3)	52.1 (49.7; 54.5)	83.0 (81.1; 85.0)
Older adults (> 65 y)	35.1 (33.2; 37.1)	17.1 (15.5; 18.5)	69.8 (67.1; 72.7)	42.5 (40.5; 44.4)	79.3 (77.5; 80.9)
**Education level**					
Up to primary (< 8 y)	39.1 (36.6; 41.5)	16.5 (14.7; 18.4)	64.9 (61.1; 68.7)	50.0 (47.4; 52.5)	82.6 (80.4; 84.7)
Secondary (8–12 y)	35.7 (34.2; 37.3)	30.8 (29.3; 32.4)	74.9 (73.0; 76.9)	53.5 (51.9; 55.3)	75.0 (73.3; 76.5)
Beyond secondary (> 12 y)	34.6 (32.2; 37.0)	33.2 (30.7; 35.8)	80.8 (78.4; 83.1)	48.0 (45.5; 50.8)	69.7 (66.8; 72.2)
**Household income**					
Lower	37.1 (34.6; 39.5)	20.4 (18.3; 22.6)	65.7 (61.8; 69.6)	53.4 (50.8; 56.1)	77.2 (74.7; 79.6)
Middle	37.6 (35.8; 39.5)	28.8 (27.2; 30.7)	73.4 (71.1; 75.6)	51.8 (49.9; 53.8)	76.8 (75.0; 78.5)
Higher	33.9 (31.4; 36.6)	33.1 (30.6; 36.1)	83.3 (80.9; 86.0)	47.7 (44.8; 50.3)	73.2 (70.6; 75.8)
**Health insurance**					
Public	37.6 (36.3; 39.1)	27.1 (25.7; 28.4)	72.9 (71.0; 74.5)	52.0 (50.5; 53.4)	77.9 (76.6; 79.1)
Private	32.6 (30.3; 34.9)	30.1 (27.9; 32.5)	79.4 (76.9; 81.7)	50.5 (48.1; 52.9)	69.6; 67.2; 72.0)
**Ethnicity indigenous**					
Yes	38.9 (35.3; 42.3)	23.8 (20.7; 26.7)	71.9 (67.2; 76.1)	60.6 (56.9; 64.3)	75.4 (71.9; 78.8)
No	36.0 (34.8; 37.2)	28.4 (27.2; 29.6)	75.2 (73.7; 76.6)	50.5 (49.2; 51.9)	75.7 (74.4; 76.9)
**Geographic area**					
Urban	35.9 (34.5; 37.2)	29.9 (28.7; 31.2)	75.0 (73.6; 76.4)	52.0 (50.7; 53.4)	74.7 (73.4; 76.0)
Rural	38.4 (35.4; 41.6)	17.1 (14.8; 19.3)	73.3 (69.2; 77.2)	49.6 (46.6; 52.7)	80.6 (77.9; 83.0)

*95% CI* confidence interval 95%, *y* years.

Table 2 shows observed and expected prevalences, as well as the ratio of observed/expected, for all combinations of the five risk factors. Of the 32 possible combinations, four had an observed/expected ratio above one, which corresponded to the co-occurrence of risk factors. Combining the five risk factors resulted in an observed/expected ratio of 1.6 (95% CI 1.1; 2.1), showing that this co-occurrence is 60% higher than expected if these risk factors were independent of each other. The highest observed/expected ratio occurred for the combination of tobacco smoking and low consumption of fruits and vegetables (observed/expected ratio: 3.0; 95% CI 2.1; 3.9).

**Table 2:** Co-occurrence of risk factors in Chilean residents.

Number and prevalence of risk factors	Absence (−) or presence (+) of risk factors					Expected prevalence (%)	Observed prevalence (%)	Observed/expected prevalence (95%CI)
	Physical inactivity	Tobacco consumption	Alcohol consumption	Low fruits and vegetables consumption	Overweight/obesity			
5 (4.7%)	**+**	**+**	**+**	**+**	**+**	2.9	4.7	***1.6 (1.1; 2.1)***
4 (20.3%)	−	**+**	**+**	**+**	**+**	5.2	6.5	1.2 (0.8; 1.4)
	**+**	-	**+**	**+**	**+**	7.6	7.7	1.0 (0.8; 1.2)
	**+**	**+**	−	**+**	**+**	1.0	1.1	1.1 (0.9; 1.3)
	**+**	**+**	**+**	−	**+**	2.8	3.0	1.1 (0.7; 1.5)
	**+**	**+**	**+**	**+**	−	0.9	2.0	***2.1 (1.3; 2.9)***
3 (34.0%)	−	−	**+**	**+**	**+**	13.4	10.6	0.8 (0.5; 1,1)
	−	**+**	−	**+**	**+**	1.8	1.7	1.0 (0.8; 1.2)
	−	**+**	**+**	−	**+**	4.8	5.7	1.2 (0.9; 1.5)
	−	**+**	**+**	**+**	−	1.7	2.9	***1.7 (1.1; 2.3)***
	**+**	−	−	**+**	**+**	2.6	2.5	1.0 (0.6; 1.4)
	**+**	−	**+**	−	**+**	7.1	6.4	0.9 (0.5; 1.3)
	**+**	−	**+**	**+**	−	2.5	1.9	0.8 (0.4; 1.2)
	**+**	**+**	−	−	**+**	0.9	0.9	1.0 (0.5; 1.5)
	**+**	**+**	−	**+**	−	0.3	0.4	1.2 (0.9; 1.5)
	**+**	**+**	**+**	−	−	0.9	1.0	1.1 (0.7; 1.4)
2 (30.4%)	**+**	**+**	−	−	−	0.3	0.3	1.0 (0.6; 1.4)
	**+**	−	**+**	−	−	2.3	1.9	0.8 (0.4; 1.2)
	**+**	−	−	**+**	−	0.8	0.6	0.7 (0.4; 1.0)
	**+**	−	−	−	**+**	2.4	2.1	0.9 (0.6; 1.2)
	−	**+**	**+**	−	−	1.6	1.7	1.1 (0.7; 1.5)
	−	**+**	−	**+**	−	0.6	1.7	***3.0 (2.1; 3.9)***
	−	**+**	−	−	**+**	1.6	1.6	1.0 (0.6; 1.4)
	−	−	**+**	**+**	−	4.3	4.1	0.9 (0.6; 1.2)
	−	−	**+**	−	**+**	12.5	11.6	0.9 (0.4; 1.3)
	−	−	−	**+**	**+**	4.5	4.8	1.1 (0.7; 1.5)
1 (9.6%)	**+**	−	−	−	−	0.8	0.9	1.2 (0.8; 1.6)
	−	**+**	−	−	−	0.5	0.3	0.6 (0.3; 0.9)
	−	−	**+**	−	−	4.0	2.7	0.7 (0.3; 1.1)
	−	−	−	**+**	−	1.5	1.3	0.9 (0.5; 1.3)
	−	−	−	−	**+**	4.2	4.4	1.0 (0.6; 1.4)
0 (1.0%)	−	−	−	−	−	1.4	1.0	0.7 (0.2; 1.2)

The presence of a risk factor is show in bold. Co-occurrence of lifestyle risk factors (O/E prevalences > 1) are displayed in bolditalics.

Only 1.0% of the participants did not present any risk factor, while 9.6%, 30.4%, 34.0%, 20.3%, and 4.7% accumulated one, two, three, four, and five risk factors, respectively. Across the entire study population, 59.0% had three or more risk factors. Co-occurrence of three or more lifestyle risk factors was significantly higher in men (OR 1.56; 95% CI 1.18; 2.04), secondary (8–12 years) education level (OR 1.59; 95% CI 1.20; 2.10), and lower household income (OR 1.39; 95% CI 1.09; 1.59). On the other hand, there was an inverse association of older adults (> 65 years [OR 0.57; 95% CI 0.41; 0.79]) and rural geographic area (OR 0.77; 95% CI 0.67; 0.89) with three or more lifestyle risk factors (Table 3).

**Table 3:** Multivarible logistic regression models (OR [95%CI]) for sociodemographic characteristics with co-occurrence of three or more risk factors for non-communicable disease in Chilean residents.

Sociodemographic characteristics	Prevalence of three or more risk factors	OR (95%CI)^a^	*p*-value
Overall	59.0	–	–
**Sex**			
Women	54.6	Ref	< 0.001
Men	66.3	1.56 (1.18; 2.04)	
**Age group**			
Younger (18–36 y)	66.0	Ref	< 0.001
Middle age (37–65 y)	64.3	1.02 (0.72; 1.44)	
Older adults (> 65 y)	48.8	0.57 (0.41; 0.79)	
**Education level**			
Beyond secondary (> 12 y)	50.5	Ref	< 0.001
Secondary (8–12 y)	60.7	1.59 (1.20; 2.10)	
Up to primary (< 8 y)	63.9	1.60 (0.95; 2.68)	
**Household income**			
Higher	52.7	Ref	0.026
Middle	59.5	1.24 (0.93; 1.67)	
Lower	63.1	1.39 (1.09; 1.59)	
**Health insurance**			
Public	59.1	Ref	0.217
Private	58.8	1.02 (0.90; 1.16)	
**Ethnicity indigenous**			
Yes	61.0	Ref	
No	58.7	0.76 (0.55; 1.04)	0.380
**Geographic area**			
Urban	59.8	Ref	< 0.001
Rural	58.7	0.77 (0.67; 0.89)	

*OR* odds ratio, *95% CI* confidence interval 95%, *Ref* reference, *y* years.

^a^Multivariable logistic regression model with risk factors for non-communicable disease (as dependent variable), adjusted for sex, age, education level, household income, health insurance, ethnicity, and geographic area.

## Discussion

In this study, we described the prevalence and co-occurrence of risk factors for NCDs and examined the co-occurrence of risk factors according to sociodemographic characteristics in Chilean residents. We found a high prevalence of co-occurrence of risk factors, with over half of the participants (59%) having three or more risk factors. From the 32 possible combinations of the five risk factors included, four co-occurrences were identified, indicating that these risk factors are not independently distributed in the population. We found that the group with all five risk factors had a prevalence 60% higher than expected, which represents a particularly high-risk group for NCDs.

The highest observed/expected ratio was observed for combination of tobacco smoking and low consumption of fruits and vegetables, which is similar from what was reported in previous studies^39,40^. Well above the global average of 25% prevalence for current smoking, Chile continues to have the highest prevalence of smoking (33.3%) in Latin America^13^. Such a prevalence is high despite the reduction observed in the 2017 CNHS in comparison to the 2010 and the 2003 (43.5%%) CNHS^13^. Chile initiated a public policy against tobacco smoking with the subscription to the 2003 World Health Organization (WHO) Framework Convention on Tobacco Control (FCTC), which was ratified by the country in 2005^41^. On the other hand, time trend data from CNHS fruits and vegetables consumption (defined as consumption of at least 5 servings per day) showed that, between 2010 and 2017, there was a similar consumption (15% in both years).

The prevalence of three or more lifestyle risk factors was more frequent in men, middle age group, those with low household income, and those with secondary education level. In our study, the proportion of three or more risk factors for NCDs was higher in men (66.3%) than women (54.6%). This finding is consistent with what has been described in other countries, where more men have combined risk factors^42–44^. This higher prevalence of co-occurrence of risk factors for NCDs could partially explain why men have a shorter life expectancy than women in Chile^45^. Regarding women, it is important to note that the single most prevalent risk factor was overweight/obesity. This is particularly alarming as obesity has been observed to be a strong risk factor for multimorbidity in adult Chilean women^46^. Furthermore, high BMI was responsible for 8.7% of all cancer cases (4394 out of 50,320 cases), with a higher proportion in women (10.4%) than in men (7.1%)^12^. Corpus uteri, kidney, and gallbladder cancers had the highest population attributable fraction for BMI in women, and kidney, gallbladder and liver cancers in men^12^. Our results also reflected a high prevalence of risk factors for NCDs among younger adults (< 37 years), particularly regarding tobacco smoking, alcohol consumption, and low consumption of fruits and vegetables.

We also found that participants with lowest household income had a higher odds of co-occurrence of three or more risk factors compared to those with high household income. These results are in line with what hass been observed in other countries or regions^47,48^. For example, adults from European countries and United States with high household income were more likely to have a healthy lifestyle (defined as achieving all five healthy behaviors), when compared to those from lower household income^47,48^. Accordingly, various studies showed that people with lower household income and education level are more likely to have a unhealthy lifestyle compared to those with higher household income or higher education level^49–52^. A systematic review also showed that men and those with greater social disadvantage show riskier health behaviours; while older age was less clearly associated with risk co-occurrence than previously indicated^49^. On the other hand, the sociodemographic characteristics associated with a lower risk of presenting three or more risk factors were: living in a rural area and older adult age group. Probably, older adults presented lower prevalence of three or more risk factors due to selection bias as people with a greater co-occurrence of risk factors may not reach older age due to premature death.

Another essential aspect to mention is that Chilean residents show higher prevalences of tobacco smoking and consumption of alcohol when compared with their South American countries^31,53^. Both behaviors are causally related to several NCDs and depend especially on public policies to reduce access so that the trend of high consumption can be reversed^54^. For instance, there is a well-known effect of availability restrictions in the case of alcohol and tobacco^31,55^. These restrictions, however, are only efficient, when they are accompanied by well-implemented public policies^56,57^. Thus, the Chilean government must start to closely discuss the improvement of these policies, aiming to fill the gaps already pointed out by studies in this country^58^.

The life expectancy of Chile increased by 2 years between 2003 and 2016^59^. This increase could be due to a number of factors, such as improvements in living standards, improved medical treatment, and public health actions^13,60^. However, some unhealthy risk factors may have counterbalanced the gain in life expectancy, particularly the increase of obesity^61^ and physical inactivity^62^. Results of our analysis indicated that all risk factors (except obesity) decrease with increasing age. In a cohort of 170,672 women and men aged 51–71 years at baseline in 1996/1997 and followed-up through 2009, adherence to the common modifiable lifestyle factors had a substantial impact on the prevention of premature deaths^63^. Selective survival, however, influences the associations between lifestyle factors and survival due to the progressive elimination of less healthy subjects from a group with an unhealthy lifestyle^64^. This selective survival, therefore, results in a weakening of the association between a lifestyle factor and survival with increasing age^64^.

The results showed the usefulness of the co-occurrence of risk factors for NCDs composite measurements, mainly because previous studies have consistently found that having three or more risk factors increase the risk of premature mortality^64,65^. For that reason, a refined version of risk factor composite scores, using standardized variables that can be used in dissimilar countries and regions, would be a benefit for national and international comparative investigations. This would be essential for policy makers, because they would be able to compare results among countries and regions, encouraging international partnership in initiatives to face mutual challenges associated with risk factors for NCDs^47^. Prevention should be a top priority for health policy and preventive care should be an indispensable part of the health care system.

Our study has some limitations. It should be considered that most of the lifestyle risk factors were based on self-reported questionnaires, which may have led to information bias, possibly underestimating the prevalence of risk factors. Another limitation is the dichotomization of the “risk/no risk” behavior required for the analysis carried out, which may have led to a loss of information. On the other hand, the large, representative sample and the inclusion of multiple risk factors are considered a strength of our analyses.

This study shows that lifestyle risk factors are often interconnected. We found a higher co-ocurrence of tobacco consumption smoking and low consumption of fruits and vegetables than expected if these risk factors were independent of each other. This convergence among risk factors needs comprehensive and integral approaches to its prevention and control^66^. Our findings of co-occurrence patterns of risk factors for NCDs can inform the development of effective preventive strategies to improve the overall health of the adult Chilean residents, which, in turn, can minimize the burden of NCDs. However, more research is needed to understand better how these co-occurrences interact simultaneously and eventually impact health and well-being. Considering the current scenario, with a high prevalence of co-occurrence of risk factors for NCDs, it is important to emphasize that policies have already been proposed at the international level. The World Health Organization Global Action Plan for the Control and Prevention of NCDs 2018-2030 has presented several practical policies to address these risk factors to decrease NCDs burden^67^.

## Conclusions

The prevalence of risk factors for NCDs is fairly high in the Chilean adult population, but specific sub-populations may warrant special attention. This novel analysis has identified important co-occurrences of three or more risk factors in the Chilean population. Furthermore, from the 32 possible combinations of the included three or more risk factors, four clusters were identified, indicating that these risk factors are not independently distributed in the population. The strong association between tobacco consumption and low fruit and vegetable consumption among adults suggests that interventions related to these substances could occur simultaneously. Thus, prevention strategies should consider the population most exposed to the main risk factors. Public health policies and interventions could consider these co-occurrences as a potential target for population stratification. Our results show possibilities for interventions related to risk factors for NCDs, but additional research on specific intervention approaches is needed to assess the impact of comprehensive actions on the reduction of co-occurrence of risk factors for NCDs.
